# Identifying routine clinical predictors of non‐adherence to second‐line therapies in type 2 diabetes: A retrospective cohort analysis in a large primary care database

**DOI:** 10.1111/dom.13865

**Published:** 2019-10-07

**Authors:** Beverley M. Shields, Andrew T. Hattersley, Andrew J. Farmer

**Affiliations:** ^1^ Institute of Biomedical and Clinical Science, University of Exeter Medical School University of Exeter Exeter UK; ^2^ Nuffield Department of Primary Care Health Sciences University of Oxford Oxford UK

**Keywords:** adherence, compliance, medical possession ratio, medication, second‐line, therapy, treatment, Type 2 diabetes

## Abstract

**Aims:**

To investigate whether combinations of routinely available clinical features can predict which patients are likely to be non‐adherent to diabetes medication.

**Materials and Methods:**

A total of 67 882 patients with prescription records for their first and second oral glucose‐lowering therapies were identified from electronic healthcare records (Clinical Practice Research Datalink). Non‐adherence was defined as a medical possession ratio (MPR) ≤80%. Potential predictors were examined, including age at diagnosis, sex, body mass index, duration of diabetes, glycated haemoglobin, Charlson index and other recent prescriptions.

**Results:**

Routine clinical features were poor at predicting non‐adherence to the first diabetes therapy (c‐statistic = 0.601 for all in combined model). Non‐adherence to the second drug was better predicted for all combined factors (c‐statistic =0.715) but this improvement was predominantly a result of including adherence to the first drug (c‐statistic =0.695 for this alone). Patients with an MPR ≤80% for their first drug were 3.6 times (95% confidence interval 3.3,3.8) more likely to be non‐adherent to their second drug (32% vs. 9%).

**Conclusions:**

Although certain clinical features were associated with poor adherence, their performance for predicting who is likely to be non‐adherent, even when combined, was weak. The strongest predictor of adherence to second‐line therapy was adherence to the first therapy. Examining previous prescription records could offer a practical way for clinicians to identify potentially non‐adherent patients and is an area warranting further research.

## 1 INTRODUCTION

Adherence to oral glucose‐lowering therapies is a major problem in diabetes, with up to two‐thirds of patients not taking their medication as prescribed.^1^, ^2^ Poor medication adherence is associated with poor glycaemic response, with patients who take <80% of their intended medication achieving half the expected reduction in glycated haemoglobin (HbA1c).^3^

Identifying which patients are likely to be non‐adherent would have clear benefits in enabling interventions aimed at improving medication adherence, and consequently, improving glycaemic control. A wide range of factors have been shown to be associated with poor adherence to diabetes medications including clinical features (female sex, younger age, non‐white ethnicity), other comorbidities such as depression,^2^ the class of medication and other prescriptions (medications after first‐line metformin,^4^ total daily pill burden),^5^, ^6^ healthcare system (insurance, medication costs)^2^, ^5^ and psychosocial factors (medication beliefs, physician trust).^6^, ^7^ In addition, adherence to previous medications has been shown to help improve predictive ability for determining adherence to statins^8^, ^9^ and, more recently, in patients with cardiometabolic disorders.^10^ Adherence differs by medication class, with adherence being poorer with first‐line metformin therapy.^4^ The time of first intensification, is therefore an appropriate time point to review adherence when considering optimal second‐line treatment.

In order to develop a practical clinical tool for identifying patients likely to be non‐adherence, it needs to be based on routinely available clinical features. Combinations of features are likely to yield greater predictive ability, but they need to be combined appropriately. Clinical prediction models, such as Q‐Risk or the Framingham risk score, provide a way of including multiple clinical predictors to determine the probability or risk of a particular outcome, and the utility of these can be increased by embedding them into routine clinical systems.

We aimed to determine whether clinical features and prescription records in routine primary care data could be combined in a clinical prediction model to help identify patients most likely to be non‐adherent to type 2 diabetes medications.

## 2 METHODS

### 2.1 Study cohort

Data were extracted on all patients starting oral glucose‐lowering drugs after January 1, 2004 registered with general practitioners (GPs) contributing to the Clinical Practice Research Datalink (CPRD). The CPRD is the largest validated primary care longitudinal health record database in the world, with data from >700 general practices throughout the UK. The dataset comprised the General Practice Online Data (CPRD GOLD), downloaded in January 2018. We did not extract data from patients treated with injectable therapies or oral solutions as either their first‐ or second‐line diabetes treatment.

A detailed explanation of our data cleaning and inclusion criteria has been published previously, including code lists.^3^ In brief, to ensure only patients with Type 2 diabetes were included, patients who had either no record of diabetes, polycystic ovary syndrome treated with metformin, or other forms of diabetes (eg, maturity‐onset diabetes of the young, steroid‐induced) were excluded using standard CPRD medcodes.^3^ As there are known coding errors with Type 1 diabetes in primary care,^11^ patients with likely type 1 diabetes were removed from the data by excluding patients with an age at diagnosis <35 years or those prescribed insulin within 1 year of diagnosis.

Patients were included in the analysis where at least 1 year of prescribing data were available and there were no gaps in registration. We removed records where patients started two drugs at the same time (2.2% of records). For patients whose first prescriptions were within 91 days of the current registration date we had no way of determining whether these were genuine new diabetes cases or whether they were continuing existing therapy, as we had no details of their previous practice records. These cases were therefore excluded.

As the focus of our analysis was on the first intensification of diabetes therapy, patients who only had records for their first‐line diabetes therapy but no subsequent therapies were excluded.

### 2.2 Calculating adherence

Adherence to the first‐ and second‐line therapies was determined by GP issue of prescriptions for glucose‐lowering medication. The medical possession ratio (MPR) was used as the metric by which to report adherence.^12^ MPR was defined as the number of days of available medication (calculated by dividing the quantity prescribed by the daily dose for each prescription) divided by the number of days between the first prescription and the adherence period end date, multiplied by 100. The adherence period end date was defined as (i) 365 days after the first prescription date (if the patient remained on the same diabetes treatment within that time period), or (ii) for patients who stopped/changed drug within a year or the prescription records ended within a year, the penultimate prescription date before the stop or change (as subsequent prescriptions are needed to determine days covered between prescriptions). In line with previous work, for MPR to be calculated, the patient needed to have at least three valid prescriptions for the drug covering at least 90 days, and not have a break between prescriptions of >6 months (as this was considered “stopping” the drug). In some instances (21% of 6 589 177 prescription records in the CPRD), the daily dose was recorded as zero. Where dose information was not available for any of the prescriptions within the adherence period, MPR was not calculated. For those where there were at least three valid prescriptions, but dose was missing from others, we removed the prescription with missing dose and the time between that prescription and the next from the denominator. An MPR <20% or > 120% was considered to reflect errors in recording of dose and so was excluded.

### 2.3 Predictors

Age at diagnosis was defined as described previously based on the age at the earliest of diabetes drug code, HbA1c in diabetes range, or prescription for glucose‐lowering medication.^3^ Baseline BMI and HbA1c are reported as the closest to the drug start date within the previous 6 months. To determine use of other non‐diabetes medications at baseline, prescriptions were extracted for patients for the 3 months prior to the drug start date. Mean number of tablets per day was calculated based on the total quantity of tablets for available prescriptions in that time period divided by 91 (quantity was chosen rather than daily dose as this was recorded more frequently). Blood pressure‐ and lipid‐lowering treatments were identified based on British National Formulary codes. The Charlson comorbidity index was calculated to determine the impact of comorbidities using previously published codes.^13^ MPR for the first drug was used as a predictor of MPR for the second drug.

### 2.4 Statistics

#### 2.4.1 Defining non‐adherence

Clinically, the most important question is how likely patients are to be non‐adherent rather than accurately trying to predict the actual adherence, particularly as the majority of patients have high adherence levels. Furthermore, the association between adherence to the first‐ and second‐line drugs was non‐linear, and initial simple models treating adherence as a continuous outcome showed poor model fit. We chose to focus on logistic regression modelling, therefore, comparing those with ≤80% MPR with those with >80% MPR. MPR≤80% was chosen to reflect non‐adherence as it is the most commonly used indicator^2^ and is the threshold below which poor adherence impacts on response.^3^

#### 2.4.2 Model development

Models to predict non‐adherence to therapy (MPR≤80%) were built using logistic regression. Changes to coefficients and model fit were checked at each stage to ensure models were robust. The shapes of the associations with the model outcome were checked by generalized additive model plots of the fits of each of the continuous predictors against the binary adherence outcome. For non‐linear associations, simple linear splines were used in regression models with knots defined at turning points seen on the plots and coefficients for the separate slopes extracted to aid interpretation of results. Adherence to the first drug was added as a predictor of non‐adherence to the second drug as a binary variable split at 80% MPR and was also treated continuously in logistic regression models. When analysed continuously MPR was capped at 100% as the association with model outcome flattened out after this point. As a final sensitivity analysis, restricted cubic splines were used for all continuous variables in full multivariable models to determine whether the fit of the models could be improved further.

Models were developed on a complete case basis. No imputation was carried out, but variables were in general available on >80% of the cohort, and models were checked for their sensitivity to missing data. Ethnicity was poorly captured, comprising only 48% of the patients, so separate models were developed including and excluding ethnicity.

Performance of the models was determined using receiver‐operating characteristic (ROC) curve analysis, the c‐statistic (equivalent to area under the ROC curve of the predicted probabilities from the logistic regression models), Somers' rank correlation (Dxy), and Nagelkerke's R^2^.

Coding of the CPRD data was carried out in stata version 14. Statistical analysis was carried out in R version 3.5.1.

### 2.5 Ethics

Approval for the study was granted by the CPRD Independent Scientific Advisory Committee (ISAC protocol 18_062).

## 3 RESULTS

A total of 198 628 patients with Type 2 diabetes had prescription records for oral glucose‐lowering drugs since 2004. Following removal of those records with too little or incomplete data there were 129 565 patients with sufficient quality data. A total of 67 882 patients had prescription records for both their first‐ and second‐line therapies and so were eligible for analysis. The 61 683 patients who only had prescription records for a first‐line therapy had a shorter period of prescriptions covered (mean 4.3 years vs. 6.7 years) and were less likely to be metformin‐treated (88% vs. 93%) than those who also had records for a second drug.

**Figure 1:**
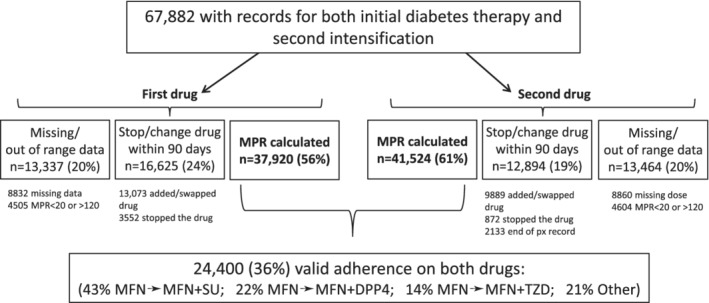
Coding of adherence category for the 67 882 patients eligible for analysis. DPP‐4, dipeptidyl peptidase‐4 inhibitors; MFN, metformin; MPR, medical possession ratio; SU, sulphonylureas; TZD, thiazolidinediones

Figure 1 shows the coding of the 67 882 patients suitable for analysis. Of these, 24 400 (36%) had a valid adherence measure on both their first and second diabetes medication (characteristics in Table S1). The characteristics of patients for whom adherence could be calculated were broadly similar to those with missing data and those who stopped/changed their treatment within 90 days (Tables S2 and S3), except missing dose data were more likely in those whose second drug was a sulphonylurea, and patients who stopped/changed their first treatment were less likely to be treated with metformin and had a higher HbA1c level.

### 3.1 Adherence to first diabetes therapy: Clinical predictors explain little variance in adherence

#### 3.1.1 Pill burden as a predictor of non‐adherence

In all, 19% of the cohort had an MPR ≤80% for their first drug. Patients who were non‐adherent to their first drug (MPR≤80%) were more likely to have a lower HbA1c, to be treated with fewer tablets per day at baseline, to have a longer diabetes duration and to be younger, and were less likely to be treated with lipid‐lowering treatment, anti‐hypertensive treatment and anti‐depressive treatment (Table S4). Those treated with metformin were more likely to be non‐adherent (MPR≤80%) than those treated with other tablets (21% vs. 11%; *P* < .0001).

#### 3.1.2 Clinical and demographic characteristics of non‐adherence

**Figure 2:**
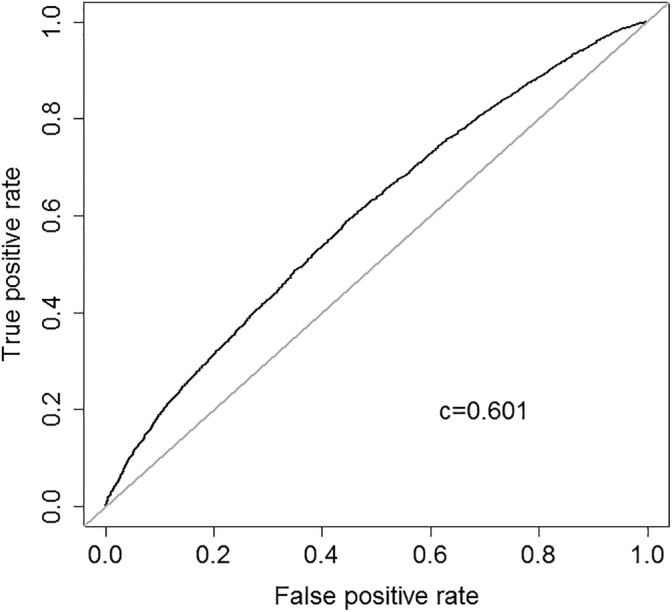
Receiver‐operating characteristic curve showing the discrimination between adherent and non‐adherent patients for the first diabetes drug when using predicted values from the full logistic regression model (c‐statistic =0.601)

In logistic regressiong models (Tables S4 [univariate] and Table S5 [multivariable]), younger age at diagnosis was associated with a decreased probability of being non‐adherent, but only up to the age of 70 years (Figure S1). Treatment with blood pressure‐lowering drugs was associated with being non‐adherent, but this association disappeared when adjusting for number of tablets per day. Coefficients for all other predictors remained similar when added into a combined multivariable logistic regression model (Table S5). Although highly significant given the large sample size, associations were weak, even with all predictions combined (Nagelkerke's R^2^ = 0.033), and the full multivariable model showed a weak correlation between predicted probabilities and outcome (Dxy = 0.2). The ROC curve of predicted values from the full logistic regression model showed poor discrimination between adherent and non‐adherent patients (c‐statistic =0.601; Figure 2).

### 3.2 Adherence to the first drug was the major predictor of adherence to the second drug

#### 3.2.1 Pill burden as a predictor of non‐adherence

**Figure 3:**
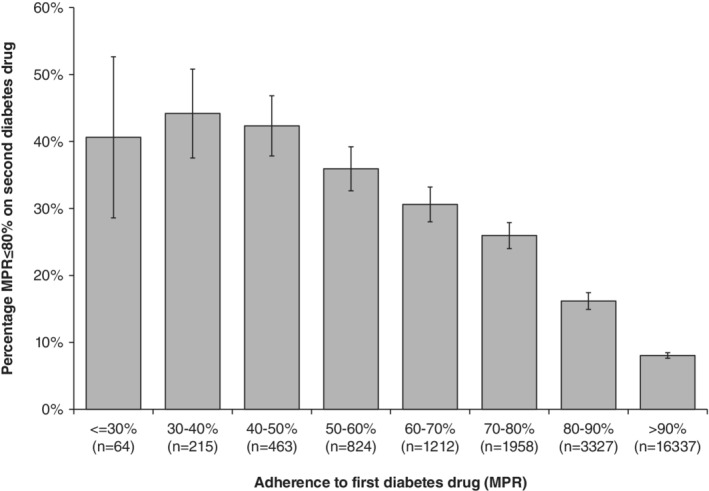
Percentage of individuals non‐adherent (medical possession ratio [MPR] ≤80%) to their second diabetes therapy based on adherence (MPR) to their first diabetes therapy. Error bars represent 95% confidence intervals

**Figure 4:**
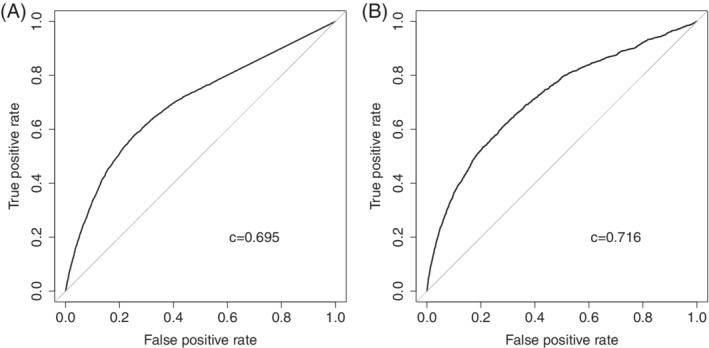
Receiver‐operating characteristic curves showing the discrimination between adherent and non‐adherent patients for the second diabetes drug when using A, adherence to the first diabetes drug alone and B, predicted values from the full logistic regression model including adherence and other baseline clinical features

Adherence to the second diabetes drug was higher than adherence to the first drug (95.5% vs. 92.5%; *P* < .0001), with fewer patients having an MPR≤80% for their second drug (14% vs. 19%; *P* < .0001). Those treated with metformin were more likely to be non‐adherent (MPR≤80%) compared with those treated with other drugs (17% vs. 13%; *P* < .0001). The differences in characteristics between adherent and non‐adherent patients for their second drug are shown in Table S6. In univariate analysis, the strongest predictor of being non‐adherent to the second drug was the MPR for the first drug (z = 37.5, Table S6). A total of 32% of those with an MPR≤80% on their first drug were non‐adherent on their second drug compared with only 9% with an MPR > 80% (relative risk 3.6 [95% confidence interval 3.3, 3.8]). Those with the lowest rates of adherence to their first drug were the most likely to be non‐adherent to their second drug (Figure 3). Adherence to drug 1, as either a binary (≤80% or > 80% MPR) or continuous predictor of adherence to the second drug had better discriminative ability than all clinical features combined for the first drug (c‐statistic =0.646 [binary] and 0.695 [continuous] vs. 0.601 [clinical features only]; Figure 4A).

#### 3.2.2 Clinical and demographic characteristics of non‐adherence

In terms of other predictors (Tables S6 [univariate] and Table S7 [multivariate]), greater number of tablets per day was associated with lower probability of being non‐adherent, with the association flattening out after 10 tablets (Figure S1). Patients who were non‐adherent were less likely to be treated with blood pressure‐ and lipid‐lowering treatment, but the associations weakened when added into logistic regression models with other features, and became non‐significant when adding in mean number of tablets per day. Use of anti‐depressant medication was associated with being non‐adherent, and this association became stronger when adding in mean number of tablets per day.

The level of HbA1c and duration of diabetes showed weak associations with adherence to second therapy, with higher HbA1c and shorter duration associated with being more likely to be non‐adherent. Similarly to the association with first diabetes therapy, younger age showed a weak association with being non‐adherent up until age 70 years, after which the association flattened out (Figure S1). Other factors including gender, body mass index and Charlson comorbidity index were not associated with adherence.

The addition of all other features in the model added little in terms of prediction and discriminative ability between adherent and non‐adherent patients over and above using adherence to the first drug alone (adherence explained 80% of the log likelihood of the full model; c‐statistic =0.716 [combined model] vs. 0.695 [adherence alone]). A full model where restricted cubic splines were fitted for all continuous variables resulted in significantly better fit (likelihood ratio χ^2^ = 86.9, *P* < .00001); however, this resulted in marginal improvement in discriminative ability between adherent and non‐adherent patients (c‐statistic =0.721), despite the increase in complexity.

When ethnicity was added to the models (Tables S8 and S9) patients of black and Asian ethnicity were found to be more likely to be non‐adherent compared with patients of white ethnicity. Coefficients remained broadly similar, and changes mainly reflected the smaller dataset rather than the addition of ethnicity to the model. Ethnicity significantly added to the full logistic regression model (likelihood ratio χ^2^ = 33, *P* < .0001 when compared with full model on only those with ethnicity recorded); however, the improvement in discrimination between adherent and non‐adherent patients was marginal (0.724 vs. 0.719 when models developed on datasets of only those with ethnicity recorded).

## 4 DISCUSSION

We found that the strongest predictor of being adherent to second‐line therapy in type 2 diabetes was a patient's adherence to their first‐line therapy. Our findings show that patients with ≤80% adherence to their first drug were nearly four times more likely to be non‐adherent to their second drug, and the risk of non‐adherence on the second drug increases as the MPR for the first drug decreases. Other routinely available clinical features, although statistically significant, offer little additional discriminative ability in identifying likely non‐adherent patients; therefore, although there are many clinical features that are associated with non‐adherence to medications as has been previously reported, most are not sufficiently predictive for clinical discrimination even when used in combination, and the only indicator with reasonable discriminative ability is recording of prescribing of medication. This is likely to relate to the fact that many predictors of adherence are social or psychological and therefore not captured by routine data.

Previous studies have identified a wide range of factors that were associated with low adherence.^2^, ^5^, ^6^, ^7^ Some of these relate to the context in which adherence is being examined. For example, one study examining a pharmacy claims database in a healthcare system where co‐payments by patients are required reported factors such as income and out‐of‐pocket costs as relevant.^5^ Other studies cite associations but do not report the extent to which these contribute to overall non‐adherence.^2^ As noted in a recent review, many of the factors identified as having an association with medication adherence are inconsistent across studies and are not modifiable.^6^ More recent studies have started to examine the relative contribution of different risk factors for non‐adherence across a range of treatments for long‐term conditions: prior non‐adherence and trajectories of use of treatment appear to be consistent and clinically relevant factors.^8^, ^9^, ^10^

There are two factors widely cited in the literature that are of particular note: depression and ethnicity. A systematic review and meta‐analysis has shown an association of depression and non‐adherence with diabetes treatment recommendations including medication use^14^; however, measurement of adherence, particularly with self‐report can be biased for some types of measure.^15^ The extent to which depression leads to non‐adherence, or whether non‐adherence to a wide range of self‐care activities leads to depression is unclear.^16^ Non‐white ethnicity is another key factor associated with non‐adherence,^17^ but the issues associated with this are complex.^7^ Although both of these factors were identified as significantly associated, they added little in terms of further discrimination in the full models. It is possible with the limited data we had that we may have underestimated their effects in our population, and in some settings these factors may be important discriminators.

Many factors relating to non‐adherence to diabetes medications are not captured in routine data. Nevertheless, it is possible that patterns of previous prescriptions issued could provide a practical indicator that clinicians could use to determine those where further assistance may be offered to improve adherence. Looking at dates when previous medications were prescribed and determining whether they are consistent with regular usage may offer a simple indicator, particularly if previous prescription coverage was considerably longer than that implied by the prescribed dose.

With the evidence provided by these data, along with other recent studies, there are now strong indications that monitoring of adherence could be of benefit. Although GP computer systems have the relevant data available and some can calculate previous MPRs based on prescription dates and prescribed daily dose, algorithms to improve data quality such as range and error checks are not in place and would be an important area for future consideration. With increasing availability of digital data, different sources of data could be incorporated to improve the accuracy with which individuals at risk might be identified, including dispensing data and home monitoring (including diaries) of taking medications. There is also a need to monitor constantly the clinical pathways through which the prescribing and dispensing of medications occur, for example, the automatic prescribing of prescriptions at regular intervals does not necessarily lead to the dispensing or collection of those medications by a patient. Prompts for medication collection by online pharmacies are already available to many patients, but linkage of data with other parts of the healthcare system is needed. Making this available to patients to inform their own decision‐making, potentially with targeted and tailored messaging around best use of medication, is an area currently being explored.^18^, ^19^

Major strengths of the present study include the large sample size and use of real‐life data, so reflecting what is currently happening in routine primary care. This meant that we were able to study simple clinical features easily available in clinical practice. By combining measures in prediction models, we have been able to move beyond simple associations to instead determine which factors are sufficiently useful to help identify potential non‐adherent patients.

The present study has a number of limitations. Routinely collected electronic health record observational data are messy and some fields are poorly captured. In particular, ~20% of prescribed doses were missing in the dataset. Furthermore, we used MPR as our outcome measure of adherence, but this is only related to prescriptions issued rather than those collected. However, it is a widely accepted measure and, although likely to underestimate overall adherence measures, offers a practical way of exploring non‐adherence in routine data. The main models presented were based on logistic regression predicting non‐adherence defined based on an MPR≤80% compared with an MPR > 80% and so some information may have been lost due to dichotomization. We chose to use this outcome as it is a commonly used indicator for non‐adherence and is the threshold below which poor adherence impacts on glycaemic response to therapy. As a sensitivity analysis, we tried more complex modelling methods and none of the approaches resulted in significant gains in predictive ability, particularly given their added complexity and the difficulty in interpreting findings. We therefore see the present paper as highlighting the importance of adherence to previous medications compared with other baseline factors, rather than producing a final prediction model for non‐adherence, which would be important to build upon in future work. The extent to which prediction might vary between medications and across different conditions remains to be explored further.

In conclusion, we have shown that the strongest predictor of adherence to second‐line therapy in type 2 diabetes is previous adherence to the first‐line therapy. Examining previous prescription records would offer a practical way for clinicians to identify potentially non‐adherent patients and is an area warranting further research.

## CONFLICT OF INTEREST

None declared.

## AUTHOR CONTRIBUTIONS

A.F., B.S. and A.H. conceived the study design. B.S. performed all analysis and wrote the initial draft. A.F. and A.H. aided interpretation of findings and edited and commented on the manuscript.

## DATA AVAILABILITY

The data were used under a licence from the CPRD. CPRD data can be obtained through submission to the CPRD Independent Scientific Advisory Committee.

## Supporting information


**Appendix S1:** Supporting information.
