# Supplementary material for: Identifying routine clinical predictors of non‐adherence to second‐line therapies in type 2 diabetes: A retrospective cohort analysis in a large primary care database
Source: Diabetes Obes Metab. 2019 Oct 7;22(1):59–65. doi: 10.1111/dom.13865 (PMC6916179; doi:10.1111/dom.13865)
Supplement: Supplementary file 1 — Appendix S1: Supporting information. [file DOM-22-59-s001.docx]

Supplementary Table 1 – Characteristics of main analysis cohort.

|  | Adherence period 1:  First diabetes drug | | Adherence period 2:  Second diabetes drug | |
| --- | --- | --- | --- | --- |
|  | N | Median (IQR) or n(%) | n | Median (IQR) or n(%) |
| Male | 24400 | 15092 (62%) |  |  |
| White ethnicity | 11727 | 10702 (91%) |  |  |
| Age at drug start (y) | 24400 | 59 (51, 68) | 24400 | 62 (54, 70) |
| Duration of diabetes (y) | 24400 | 0.48 (0.04, 2.43) | 24400 | 3.6 (1.8, 6.0) |
| BMI (kg/m^2^) | 19289 | 32 (28, 36) | 17176 | 32 (28, 36) |
| Mean no. tabs per day* | 21697 | 3.3 (1.3, 6.4) | 24207 | 6.8 (4.6, 10.3) |
| HbA1c (mmol/mol) | 20221 | 69 (60, 87) | 20003 | 67 (61, 78) |
| On blood pressure lowering treatment* | 21708 | 14096 (65%) | 24232 | 17390 (72%) |
| On lipid lowering treatment* | 21708 | 11485 (53%) | 24232 | 18796 (78%) |
| On anti-depressant* | 21708 | 3467 (16%) | 24232 | 4285 (18%) |
| Charlson Index | 24400 | 1 (1, 2) | 24400 | 2 (1, 3) |

*as determined by prescriptions in 3 months preceding the start date of the diabetes drug

Supplementary Table 2 – Characteristics of cohort by adherence category for first- and second-line diabetes glucose lowering therapy. Data presented as median (inter-quartile) range for continuous variables or percentages for categorical variables.

|  | Missing/erroneous data | Stop/change drug* | Adherence calculated |
| --- | --- | --- | --- |
| **Drug 1 (First-line)** |  |  |  |
| N | 13337 | 16625 | 37920 |
| Male (%) | 57.7% | 56.9% | 60.7% |
| Age at diagnosis (y) | 59 (50, 67) | 59 (50, 69) | 58 (50, 66) |
| BMI (kg/m^2^) | 31.5 (27.9, 36.0) | 30.8 (27.1, 35.2) | 31.8 (28.1, 36.2) |
| Duration of diabetes (y) | 0.4 (0.03, 2.4) | 0.3 (0.01, 2.3) | 0.5 (0.04, 2.4) |
| HbA1c (mmol/mol) | 70 (61, 90) | 75 (61, 99) | 68 (60, 86) |
| Drug class |  |  |  |
| MFN | 88.3% | 83.5% | 90.6% |
| SU | 11.2% | 15.2% | 8.5% |
| Other | 0.5% | 1.3% | 0.9% |
| **Drug 2 (Second line)** |  |  |  |
| N | 13464 | 10761 | 41524 |
| Male | 58% | 56% | 60% |
| Age at diagnosis (y) | 59 (51, 68) | 58 (49, 67) | 58 (50, 67) |
| BMI (kg/m^2^) | 30.8 (27.3, 35.0) | 31.0 (27.3, 35.5) | 31.2 (27.6, 35.6) |
| Duration diabetes (y) | 3.0 (1.1, 5.6) | 3.0 (1.2, 5.7) | 3.0 (1.2, 5.5) |
| HbA1c (mmol/mol) | 68 (61, 83) | 69 (60, 85) | 68 (61, 81) |
| Drug class |  |  |  |
| MFN | 20.3% | 21.3% | 21.9% |
| SU | 63.1% | 50.6% | 46.6% |
| DPP4 | 9.2% | 15.4% | 18.3% |
| TZD | 5.5% | 9.3% | 11.0% |
| Other | 1.8% | 3.3% | 2.2% |

*those that stopped due to end of prescription records not included. P values not presented as large sample size means even very small differences are highly statistically significant

Supplementary Table 3 - Characteristics of cohort when starting first diabetes therapy for those who have adherence calculated on both drugs compared with those who do not. Duration of diabetes and HbA1c represent skewed data so descriptives presented as median and interquartile range for consistency across all. P values not presented as large sample size means even very small differences are highly statistically significant

|  | Adherence calculated on both drugs | Adherence not calculated on both drugs |
| --- | --- | --- |
| N | 24400 | 43482 |
| Male (%) | 62% | 58% |
| Age at diagnosis (y) | 58 (50, 66) | 59 (50, 68) |
| BMI (kg/m^2^) | 31.9 (28.3, 36.4) | 31.2 (27.6, 35.7) |
| Duration of diabetes (y) | 0.5 (0.4, 2.4) | 0.4 (0.02, 2.4) |
| HbA1c (mmol/mol) | 69 (60, 87) | 71 (60, 91) |
| Drug class |  |  |
| MFN | 91% | 87% |
| SU | 8% | 12% |
| Other | 1% | 1% |

Supplementary Table 4 – Comparison of patient characteristics between non-adherent and adherent patients for drug 1. Data presented as % or median (IQR) and results from logistic regression of each variable predicting non-adherence. β (SE) represents the log odds ratio of being non-adherent per unit increase in the predictor and its associated standard error, z is the test statistic (β/SE), OR (95% CI) is the odds ratio for being non-adherent per unit increase in the predict and the associated 95% confidence interval, p is the p value. Positive β coefficients and odds ratios >1 represent higher likelihood of being non-adherent as the predictor increases.

|  | Median (IQR) or % | | Univariate logistic regression predicting non-adherence to first diabetes drug | | | |
| --- | --- | --- | --- | --- | --- | --- |
|  | Adherence ≤80% for first drug  (n=4736) | Adherence >80% for first drug  (n=19664) | β (SE) | z | OR (95% CI) | p |
| Male gender | 63% | 62% | 0.046 (0.03) | 1.4 | 1.05 (0.98, 1.12) | 0.2 |
| Age at drug start (y) † | 57 (49, 65) | 60 (52, 68) | -0.03 (0.002) *(if <70)* | -13.9 | 0.972 (0.968, 0.976) | <0.0001 |
|  |  |  | 0.016 (0.006) *(if>70)* | 2.6 | 1.016 (1.00, 1.03) | 0.01 |
| Duration of diabetes (y) | 0.7 (0.06, 2.6) | 0.4 (0.03, 2.4) | 0.028 (0.006) | 4.7 | 1.029 (1.017, 1.041) | <0.0001 |
| BMI (kg/m^2^) | 32.2 (28.6, 36.9) | 32.0 (28.0, 36.0) | 0.011 (0.003) | 4.1 | 1.011 (1.006, 1.017) | <0.0001 |
| Mean no of tablets per day* | 2.7 (1.2, 5.3) | 3.5 (1.4, 6.6) | -0.053 (0.004) | -11.9 | 0.948 (0.940, 0.956) | <0.0001 |
| HbA1c (mmol/mol) | 68.0 (59.6, 83.6) | 69.0 (61.0, 88.0) | -0.006 (0.0009) | -6.6 | 0.994 (0.992, 0.996) | <0.0001 |
| On BP lowering treatment* | 60% | 66% | -0.238 (0.04) | -6.7 | 0.788 (0.735, 0.845) | <0.0001 |
| On lipid lowering treatment* | 48% | 54% | -0.241 (0.035) | -7.0 | 0.786 (0.734, 0.841) | <0.0001 |
| On anti-depressant* | 15% | 16% | -0.092 (0.048) | -1.9 | 0.912 (0.829, 1.002) | 0.06 |
| Charlson Index † | 1 (1, 2) | 1 (1, 2) | -0.053 (0.016) | -3.4 | 0.948 (0.919, 0.978) | 0.0008 |

*as determined by prescriptions in 3 months preceding the start date of the diabetes drug

† age at drug start non-linear (see supplementary figure 1): slopes of two regression lines presented for ease of interpretation

Supplementary Table 5: Coefficients from full multivariable logistic regression model of predictors of non-adherence to first diabetes drug. β (SE) represents the log odds ratio of being non-adherent per unit increase in the predictor and its associated standard error, z is the test statistic (β/SE), OR (95% CI) is the odds ratio for being non-adherent per unit increase in the predict and the associated 95% confidence interval, p is the p value. Positive β coefficients and odds ratios >1 represent higher likelihood of being non-adherent as the predictor increases.

|  | β (SE) | z | OR (95% CI) | p |
| --- | --- | --- | --- | --- |
| Male gender | 0.002 (0.044) | 0.04 | 1.002 (0.919, 1.093) | 0.97 |
| Age at drug start (y) (if <70) | -0.019 (0.003) | -6.7 | 0.982 (0.976, 0.987) | <0.0001 |
| Age at drug start (y) (if >70) | 0.021 (0.008) | 2.6 | 1.021 (1.005, 1.037) | 0.02 |
| Duration of diabetes (y) | 0.045 (0.008) | 5.5 | 1.046 (1.029, 1.063) | <0.0001 |
| BMI (kg/m2) | 0.012 (0.003) | 3.6 | 1.012 (1.006, 1.019) | <0.0001 |
| Mean no of tablets per day* | -0.056 (0.007) | -8.2 | 0.946 (0.933, 0.958) | <0.0001 |
| HbA1c (mmol/mol) | -0.009 (0.001) | -7.5 | 0.991 (0.988, 0.993) | <0.0001 |
| On BP lowering treatment* | -0.072 (0.05) | -1.5 | 0.930 (0.844, 1.0126) | 0.15 |
| On lipid lowering treatment* | -0.17 (0.05) | -3.7 | 0.842 (0.769, 0.923) | 0.0002 |
| On anti-depressant* | 0.030 (0.06) | 0.5 | 1.030 (0.908, 1.168) | 0.6 |
| Charlson Index | 0.034 (0.02) | 1.6 | 1.034 (0.992, 1.077) | 0.11 |

*as determined by prescriptions in 3 months preceding the start date of the diabetes drug

† age at drug start non-linear (Supplementary figure 1) so 2 separate slopes shown for before and after 70 for ease of interpretation

Supplementary Table 6 - Comparison of patient characteristics between non-adherent and adherent patients for second drug. Data presented as % or median (IQR) and results from univariate logistic regression of each variable predicting non-adherence. β (SE) represents the slope (log odds ratio of being non-adherent per unit increase in the predictor) and its associated standard error, z is the test statistic (β/SE), OR (95% CI) is the odds ratio for being non-adherent per unit increase in the predict and the associated 95% confidence interval, p is the p value. Positive β coefficients and odds ratios >1 represent higher likelihood of being non-adherent as the predictor increases.

|  | Median (IQR) or % | | Univariate logistic regression predicting non-adherence to second diabetes drug | | | |
| --- | --- | --- | --- | --- | --- | --- |
|  | Adherence ≤80% on second drug  (n=3265) | Adherence >80%  on second drug  (n=20406) | β (SE) | z | OR (95% CI) | p |
| MPR<80% on drug 1 (%) | 32% | 9% | 1.49 (0.040) | 37.5 | 4.42 (4.09, 4.78) | <0.0001 |
| Adherence to drug 1 (MPR) | 83.8 (66.7, 98.1) | 98.1 (87.7, 104.3) | -0.038 (0.001) | -39.0 | 0.963 (0.961, 0.964) | <0.0001 |
| Male gender | 62% | 62% | 0.016 (0.038) | 0.4 | 1.016 (0.942, 1.095) | 0.7 |
| Age at drug start (y) † | 59 (51, 68) | 62 (55, 71) | -0.029 (0.002) *(if age<70)* | -14.0 | 0.971 (0.967, 0.975) | <0.0001 |
|  |  |  | 0.024 (0.007) (*if age>70)* | 3.5 | 1.024 (1.010, 1.038) | 0.001 |
| Duration of diabetes (y) | 3.3 (1.6, 5.6) | 3.6 (1.9, 6.1) | -0.030 (0.006) | -5.0 | 0.971 (0.959, 0.982) | <0.0001 |
| BMI (kg/m^2^) | 31.4 (27.7, 36.2) | 32.0 (28.0, 36.0) | 0.028 (0.003) | 0.8 | 1.003 (0.996, 1.010) | 0.8 |
| Mean no of tablets per day† | 5.5 (3.5, 8.4) | 7.1 (4.6, 10.6) | -0.149 (0.008) *(if <10)* | -19.3 | 0.862 (0.849, 0.875) | <0.0001 |
|  |  |  | 0.006 (0.008) *(if >10)* | 0.72 | 1.006 (0.990, 1.021) | 0.3 |
| HbA1c (mmol/mol) | 69.4 (61.9, 82.0) | 66 (60, 77) | 0.012 (0.001) | 9.9 | 1.012 (1.010, 1.015) | <0.0001 |
| On BP lowering therapy* | 65% | 73% | -0.343 (0.040) | -8.6 | 0.709 (0.656, 0.767) | <0.0001 |
| On lipid lowering therapy* | 71% | 79% | -0.413 (0.042) | -9.8 | 0.661 (0.609, 0.718) | <0.0001 |
| On anti-depressant* | 19% | 17% | 0.095 (0.048) | 2.0 | 1.099 (0.999, 1.207) | 0.052 |
| Charlson Index | 1 (1, 3) | 2 (1, 3) | -0.070 (0.014) *(if<5)* | -5.0 | 0.932 (0.907, 0.958) | <0.0001 |

*as determined by prescriptions in 3 months preceding the start date of the diabetes drug

† age at drug start and mean no. of tablets per day non-linear (supplementary figure 1): slopes of two regression lines presented for ease of interpretation.

Supplementary Table 7: Coefficients from full multivariable logistic regression model of predictors of non-adherence to second diabetes drug. β (SE) represents the log odds ratio of being non-adherent per unit increase in the predictor and its associated standard error, z is the test statistic (β/SE), OR (95% CI) is the odds ratio for being non-adherent per unit increase in the predict and the associated 95% confidence interval, p is the p value. Positive β coefficients and odds ratios >1 represent higher likelihood of being non-adherent as the predictor increases.

|  | β (SE) | z | OR (95% CI) | p |
| --- | --- | --- | --- | --- |
| Adherence to first drug (%) | -0.038 (0.001) | -27.4 | 0.963 (0.960, 0.965) | <0.0001 |
| Male gender | -0.088 (0.051) | -1.7 | 0.916 (0.829, 1.013) | 0.1 |
| Age at drug start (y) (if <70) † | -0.012 (0.003) | -3.7 | 0.989 (0.980, 0.995) | 0.0002 |
| Age at drug start (y) (if >70) | 0.015 (0.010) | 1.6 | 1.016 (0.997, 1.035) | 0.1 |
| Duration of diabetes (y) | -0.013 (0.008) | -1.6 | 0.987 (0.971, 1.003) | 0.1 |
| BMI (kg/m2) | -0.005 (0.004) | -1.2 | 0.995 (0.987, 1.003) | 0.2 |
| Mean no of tablets per day (if<10) † | -0.116 (0.012) | -10.0 | 0.890 (0.870, 0.911) | <0.0001 |
| Mean no of tablets per day (if>10) | -0.007 (0.010) | -0.7 | 0.993 (0.973, 1.013) | 0.3 |
| HbA1c (mmol/mol) | 0.006 (0.002) | 3.8 | 1.006 (1.003, 1.009) | <0.0001 |
| On BP lowering treatment* | 0.099 (0.059) | 1.7 | 1.104 (0.984, 1.239) | 0.09 |
| On lipid lowering treatment* | -0.059 (0.059) | -1.0 | 0.942 (0.840, 1.058) | 0.3 |
| On anti-depressant* | 0.355 (0.068) | 5.3 | 1.426 (1.248, 1.626) | <0.0001 |
| Charlson Index | 0.005 (0.02) | 0.2 | 1.005 (0.966, 1.044) | 0.8 |

*as determined by prescriptions in 3 months preceding the start date of the diabetes drug

† age at drug start and mean no of tablets per day non-linear (supplementary figure 1) so 2 separate slopes shown for before and after 70 for ease of interpretation

Supplementary Table 8: Coefficients from full multivariable logistic regression model of predictors of non-adherence to first diabetes drugs with ethnicity included. Models based on data from 7213 individuals (1344 (19%) non-adherent, 5869 adherent). β (SE) represents the log odds ratio of being non-adherent per unit increase in the predictor and its associated standard error, z is the test statistic (β/SE), OR (95% CI) is the odds ratio for being non-adherent per unit increase in the predict and the associated 95% confidence interval, p is the p value. Positive β coefficients and odds ratios >1 represent higher likelihood of being non-adherent as the predictor increases.

|  | β (SE) | z | OR (95% CI) | p |
| --- | --- | --- | --- | --- |
| Ethnicity: White (reference category) |  |  |  |  |
| Black | 0.763 (0.205) | 3.7 | 2.15 (1.42, 3.18) | 0.0002 |
| Asian | 0.517 (0.134) | 3.8 | 1.67 (1.28, 2.16) | 0.0001 |
| Other | 0.296 (0.250) | 1.2 | 1.35 (0.81, 2.16) | 0.2 |
| Male gender | -0.02 (0.064) | -0.3 | 0.980 (0.864, 1.113) | 0.8 |
| Age at drug start (y) (if <70) | -0.013 (0.004) | -3.3 | 0.987 (0.979, 0.995) | 0.001 |
| Age at drug start (y) (if >70) | 0.023 (0.012) | 1.9 | 1.02 (1.000, 1.064) | 0.06 |
| Duration of diabetes (y) | 0.048 (0.012) | 4.0 | 1.049 (1.024, 1.074) | <0.0001 |
| BMI (kg/m^2^) | 0.009 (0.005) | 1.8 | 1.009 (0.999, 1.020) | 0.07 |
| Mean no of tablets per day | -0.054 (0.010) | -5.3 | 0.948 (0.929, 0.966) | <0.0001 |
| HbA1c (mmol/mol) | -0.007 (0.002) | -4.1 | 0.993 (0.990, 0.996) | <0.0001 |
| On blood pressure lowering treatment* | -0.082 (0.07) | -1.1 | 0.922 (0.800, 1.063) | 0.3 |
| On lipid lowering treatment* | -0.189 (0.07) | -2.8 | 0.828 (0.724, 0.946) | 0.006 |
| On anti-depressant* | 0.017 (0.09) | 0.2 | 1.017 (0.845, 1.221) | 0.9 |
| Charlson Index ⱡ | -0.002 (0.03) | -0.07 | 0.998 (0.939, 1.059) | 0.9 |

*as determined by prescriptions in 3 months preceding the start date of the diabetes drug

† age at drug start non-linear so 2 separate slopes shown for before and after 70 for ease of interpretation

ⱡ Charlson Index showed linear association in full multivariable model in contrast to univariate analysis

Supplementary Table 9: Coefficients from full multivariable logistic regression model of predictors of non-adherence to second diabetes drugs with ethnicity included. Models based on 8051 individuals (1037 (13%) non-adherent, 7014 adherent). β (SE) represents the log odds ratio of being non-adherent per unit increase in the predictor and its associated standard error, z is the test statistic (β/SE), OR (95% CI) is the odds ratio for being non-adherent per unit increase in the predict and the associated 95% confidence interval, p is the p value. Positive β coefficients and odds ratios >1 represent higher likelihood of being non-adherent as the predictor increases.

|  | β (SE) | z | OR (95% CI) | p |
| --- | --- | --- | --- | --- |
| Ethnicity: White (reference category) |  |  |  |  |
| Black | 1.087 (0.211) | 5.1 | 2.967 (1.946, 4.466) | <0.0001 |
| Asian | 0.494 (0.143) | 3.5 | 1.639 (1.233, 2.159) | 0.0005 |
| Other | 0.635 (0.249) | 2.6 | 1.887 (1.136, 3.027) | 0.01 |
| Adherence to first drug (%) | -0.037 (0.002) | -18.1 | 0.964 (0.960, 0.968) | <0.0001 |
| Male gender | -0.212 (0.07) | -2.9 | 0.809 (0.701, 0.935) | 0.004 |
| Age at drug start (y) (if <70) | -0.010 (0.005) | -2.3 | 0.990 (0.981, 0.999) | 0.02 |
| Age at drug start (y) (if >70) | -0.003 (0.015) | -0.2 | 0.997 (0.968, 1.027) | 0.4 |
| Duration of diabetes (y) | -0.023 (0.012) | -1.9 | 0.978 (0.954, 1.001) | 0.06 |
| BMI (kg/m^2^) | -0.002 (0.006) | -0.3 | 0.998 (0.987, 1.010) | 0.8 |
| Mean no of tablets per day (if<10) | -0.090 (0.017) | -5.4 | 0.914 (0.884, 0.944) | <0.0001 |
| Mean no of tablets per day (if>10) | -0.02 (0.015) | -1.3 | 0.981 (0.951, 1.010) | 0.2 |
| HbA1c (mmol/mol) | 0.007 (0.002) | 3.3 | 1.007 (1.003, 1.011) | 0.001 |
| On blood pressure lowering treatment* | 0.036 (0.085) | 0.4 | 1.037 (0.879, 1.225) | 0.7 |
| On lipid lowering treatment* | -0.205 (0.084) | -2.4 | 0.814 (0.691, 0.961) | 0.01 |
| On anti-depressant* | 0.393 (0.097) | 4.1 | 1.481 (1.224, 1.788) | <0.0001 |
| Charlson Index | 0.026 (0.029) | 0.9 | 1.027 (0.970, 1.086) | 0.4 |

*as determined by prescriptions in 3 months preceding the start date of the diabetes drug, † age at drug start and mean no of tablets per day non-linear so 2 separate slopes shown for before and after 70 for ease of interpretation

Supplementary Figure 1 – Generalised additive modelling plots showing the non-linear associations of a) age when commencing first diabetes therapy with non-adherence to the first diabetes drug, and each of b) age when commencing second diabetes therapy and c) mean number of tablets per day in the 3 months preceding the second diabetes drug, with non-adherence (MPR<80%) to the second diabetes drug. As the outcome is binary and main analysis by logistic regression the y axis is centred and on the log odds scale, so the values are not informative for interpretation but allow observation of the shape for modelling. Solid line represents fit of the data, and dashed line represents 95% confidence intervals.

| 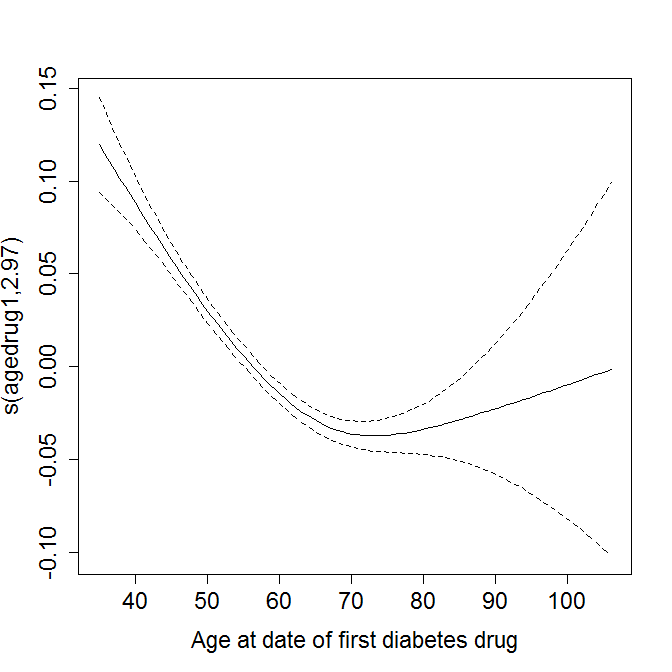 | 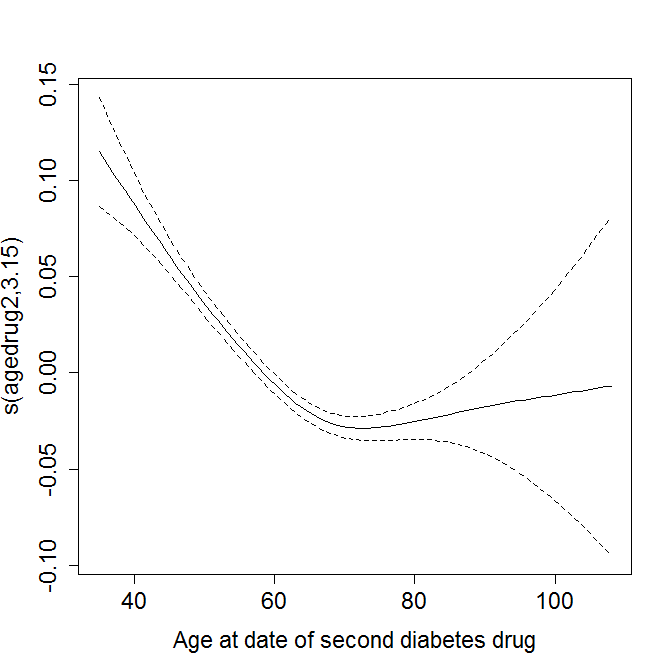 |
| --- | --- |
| 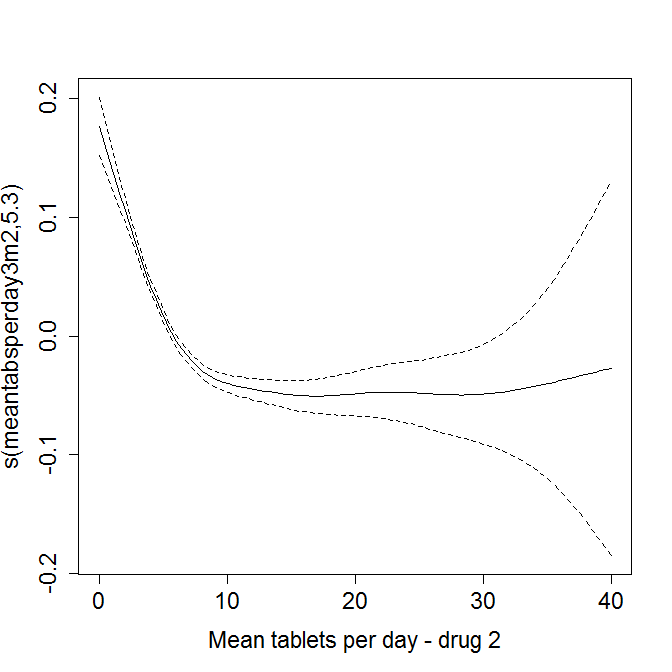 |  |
